# Causal Learning: Monitoring Business Processes Based on Causal Structures

**DOI:** 10.3390/e26100867

**Published:** 2024-10-15

**Authors:** Fernando Montoya, Hernán Astudillo, Daniela Díaz, Esteban Berríos

**Affiliations:** 1 Nexus Payment Systems SpA, Santiago 8320123, Chile; 2 Campus Casa Central, Universidad Técnica Federico Santa María, Valparaíso 2390123, Chile; 3 Fundación Instituto Profesional Duoc UC, Santiago 8240000, Chile; 4 Instituto de Tecnología para la Innovación en Salud y Bienestar, Universidad Andres Bello, Viña del Mar 2530958, Chile; 5 IT Solution SpA, Santiago 8320000, Chile

**Keywords:** causal graph, causal attribution of anomalies, causal attribution of distributional change, business process monitoring, business process mining

## Abstract

Conventional methods for process monitoring often fail to capture the causal relationships that drive outcomes, making hard to distinguish causal anomalies from mere correlations in activity flows. Hence, there is a need for approaches that allow causal interpretation of atypical scenarios (anomalies), allowing to identify the influence of operational variables on these anomalies. This article introduces (*CaProM*), an innovative technique based on causality techniques, applied during the planning phase in business process environments. The technique combines two causal perspectives: *anomaly attribution* and *distribution change attribution*. It has three stages: (1) process events are collected and recorded, identifying flow instances; (2) causal learning of process activities, building a directed acyclic graphs (DAGs) represent dependencies among variables; and (3) use of DAGs to monitor the process, detecting anomalies and critical nodes. The technique was validated with a industry dataset from the banking sector, comprising 562 activity flow plans. The study monitored causal structures during the planning and execution stages, and allowed to identify the main factor behind a major deviation from planned values. This work contributes to business process monitoring by introducing a causal approach that enhances both the *interpretability* and *explainability of anomalies*. The technique allows to understand which specific variables have caused an atypical scenario, providing a clear view of the causal relationships within processes and ensuring greater accuracy in decision-making. This causal analysis employs cross-sectional data, avoiding the need to average multiple time instances and reducing potential biases, and unlike time series methods, it preserves the relationships among variables.

## 1. Introduction

The monitoring of business processes has become a critical challenge in modern management due to the dynamic environment, increasing complexity, and interconnection of activity flows [1]. These factors demand methods that go beyond simple metric evaluations. Traditionally, such oversight has relied on static models, predefined metrics, and comparisons of established execution patterns, which limit the ability to identify and understand the true underlying causal relationships in business processes. In this context, there is a need for an approach that facilitates not only the observation but also the interpretation and explanation of business process behavior from a causal perspective.

Despite advances in predictive [2,3,4], as well as prescriptive [5,6], analysis techniques for process monitoring, such techniques are still primarily based on patterns of correlation or association, without delving into causal reasoning. This limits the ability to identify the true underlying structures that govern operations. While correlation can indicate relationships between variables, it does not necessarily establish a cause–effect relationship. Without a methodology that explains the implications of causal effects, the understanding of operational dynamics remains incomplete, which can lead to biased decisions and hinder effective problem solving. This underscores the need for a causal approach that identifies causes and interprets their implications, improving decision making in complex business environments. Throughout a business process flow, multiple distinct instances may arise due to the diversity of operational objectives. This results in varied and often complex causal relationships. Therefore, it is essential to implement advanced approaches that go beyond traditional monitoring. These approaches must be capable of interpreting and thoroughly explaining the variations in business processes, grounded in a solid causal structure.

The remainder of this paper is structured as follows: Section 2 formalizes the problem; Section 3 surveys related work; Section 4 explains the proposed technique; Section 5 presents the case study; Section 6 discusses the results; and Section 7 summarizes and concludes.

## 2. Context and Problem

This section formalizes the description of the research problem as one of causal reasoning on business processes.

### 2.1. Problem Description

**Figure 1:**
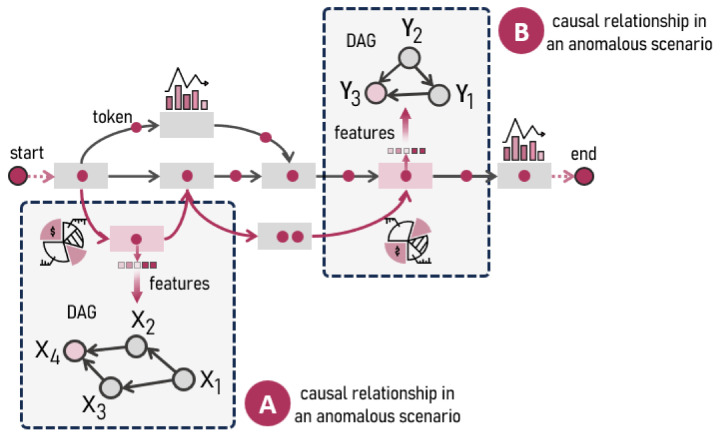
Characterization of causal variables in business process activities during anomalous scenarios with respect to the effect variables exhibiting anomalies.

Figure 1 illustrates a business process flow in which execution instances are represented as tokens corresponding to specific tasks defined in the planning of various projects. In this context, organizational areas continuously monitor a series of key performance indicators (KPIs) to manage and optimize the processes. These KPIs include compliance with service-level agreements (SLAs); resource utilization; and other critical factors, such as hours allocated to specific tasks, personnel assigned to essential roles, and the achievement of departmental objectives [7]. This systematic approach enables the measurement of anomalies in the process flow, based on the defined metrics, which manifest in critical scenarios such as those described in *A* and *B*.

Interpreting and explaining the causal relationships in these scenarios presents a considerable challenge, as it involves uncovering the underlying causal relationships connecting the various events and metrics involved. Moreover, understanding the causal direction of the variables that trigger these events is crucial to ensuring more precise and proactive process management, allowing for timely adjustments in resource allocation and task planning.

Approaching this analysis from a causal perspective establishes the directed acyclic graph (DAG) as a key tool for understanding the connections between variables [8,9], as DAGs not only aid in identifying the causes of anomalous events but also provide a more comprehensive understanding of the operational interactions within the process. Nevertheless, their implementation demands a deep understanding of causal topology. In Figure 1, the DAGs from scenarios *A* and *B* reveal different levels of complexity, where, within the same process, the operational aspects of two distinct activities are represented by the variables $X_{i}$ and $Y_{i}$, respectively. Both significantly influence the overall flow, as their interactions can determine both the stability and performance of the process, potentially leading to anomalous scenarios in the business process flow.

### 2.2. Causal Reasoning in the Business Process Context

Business process monitoring not only entails supervising key indicators but also examining how the relationships between variables influence overall performance. Understanding these relationships from a causal perspective is crucial for improving decision making and optimizing operational performance. Through causal learning, it is possible to identify how variables interact and impact the process flow, enabling early detection and correction of anomalies. Scenarios *A* and *B* (see Figure 1) illustrate how this causal approach can influence different activities within the same business process, given the distinct causal topologies between variables $X_{i}$ and $Y_{i}$.

In Scenario *A*, variables $X_{1}$, $X_{2}$, $X_{3}$, and $X_{4}$ are part of a specific activity within the process, where $X_{1}$ directly influences $X_{2}$ and $X_{3}$ and these two, in turn, affect $X_{4}$. Applying causal learning shows that $X_{2}$ and $X_{3}$ are independent of each other when conditioned on $X_{1}$, which is expressed as $X_{2}⊥\;\;\;⊥X_{3}|X_{1}$; however, when conditioned on $X_{4}$, which acts as a collider, $X_{2}$ and $X_{3}$ become dependent. This understanding introduces important considerations for business process decision making because if an anomaly occurs in $X_{4}$, causal learning identifies that the potential causes lie in $X_{2}$ and $X_{3}$. Due to the conditional independence between these variables given $X_{1}$, it is possible to analyze each one separately to determine which is contributing to the anomaly, facilitating more effective mitigation by allowing interventions to be specifically directed at the problematic variable without unnecessarily affecting other parts of the process.

On the other hand, in scenario *B*, corresponding to a different activity within the same process flow, the variables $Y_{1}$, $Y_{2}$, and $Y_{3}$ interact differently. $Y_{2}$ directly influences both $Y_{1}$ and $Y_{3}$, and $Y_{1}$ also influences $Y_{3}$. This creates a causal chain where $Y_{2}$ affects $Y_{3}$ both directly and indirectly through $Y_{1}$, indicating that all influences must be considered when analyzing $Y_{3}$. Causal learning indicates that an anomaly in $Y_{3}$ could be the result of multiple inter-related factors, requiring decision makers to account for both the direct and indirect influences of $Y_{2}$ and $Y_{1}$. Mitigating the anomaly, therefore, requires a comprehensive approach addressing all the variables involved to identify and resolve the root cause of the problem.

These scenarios highlight the importance of having previous causal insights and mechanisms for interpreting anomalies using DAGs. Without a thorough understanding of the causal relationships and dependency topology in business processes, decision making can become inefficient. Implementing causal learning and developing tools that interpret anomalies according to the causal structure allow for the identification of underlying causes and the design of effective interventions. This approach not only facilitates the resolution of current anomalies but also helps to prevent future incidents, ultimately improving the overall performance of the business.

### 2.3. Research Questions

This work proposes a monitoring methodology based on two causal perspectives, anomaly attribution and distributional change attribution [10,11]. This approach allows for identifying the contributions of each node to the observed anomalies and changes in the system’s causal mechanisms, facilitating a deep understanding of the variations and their underlying causes.

#### 2.3.1. Anomaly Attribution

The purpose of these questions is to evaluate how the causal connections between variables explain the severity of observed anomalies [10].

- ${\mathrm{RQ}}_{1}$: *What is the causal contribution of a specific variable (e.g., $X_{1}$ or $Y_{2}$) to the anomaly observed in a target variable (e.g., $X_{4}$ or $Y_{3}$), taking into account the entire causal graph?*
  The influence of variables like $X_{1}$ or $Y_{2}$ is key to understanding the origin of anomalous deviations in the system, allowing for the identification of critical points that generate these unexpected behaviors.
- ${\mathrm{RQ}}_{2}$: *How do causal paths, such as $X_{1}\to X_{2}\to X_{4}$ or $Y_{2}\to Y_{1}\to Y_{3}$, affect the severity of the anomalies observed in $X_{4}$ and $Y_{3}$?*
  Causal paths, such as $X_{1}\to X_{2}\to X_{4}$ or $Y_{2}\to Y_{1}\to Y_{3}$, break down the internal dynamics of the system, revealing how intermediate interactions contribute to the final magnitude of the observed anomaly.

#### 2.3.2. Distributional Change Attribution

The focus of these questions is to analyze how changes in causal mechanisms and interactions between nodes affect the observed distributions in different scenarios [11,12].

- ${\mathrm{RQ}}_{3}$: When comparing two different contexts, *how can we identify and quantify the changes in the distributions of target variables (e.g., $X_{4}$ or $Y_{3}$), and what evidence suggests that these differences are due to alterations in the causal mechanisms of specific variables (e.g., $X_{1}$ or $Y_{2}$)?*
  This question aims to identify which nodes experience changes in their mechanisms and how they impact the distribution of the target variables under different conditions.
- ${\mathrm{RQ}}_{4}$: *How do changes in causal interactions within paths $X_{1}\to X_{2}\to X_{4}$ and $Y_{2}\to Y_{1}\to Y_{3}$ impact the distributional properties of $X_{4}$ and $Y_{3}$?*
  This question examines how modifications in the causal relationships within specific paths alter the distributions of variables $X_{4}$ and $Y_{3}$.

## 3. Related Work

Recent literature on business process monitoring has explored causal approaches to improving efficiency and effectiveness in management. Traditionally, causal inference for estimation of effects has largely depended on expert knowledge, which may introduce potential limitations and biases in the analysis. However, there is a growing need to apply causal learning techniques to enable the autonomous discovery of causal relationships.

For example, Shoush et al. [13] used algorithms such as orthogonal random forests (**ORF**) to optimize resource allocation and intervention policies, leveraging historical data to predict needs in credit origination. This approach is complemented by the work described by Bozorgi et al. [14], which combines causal inference with reinforcement learning to enhance treatment policies, maximizing net benefits in business processes. Both studies emphasize the importance of accurately evaluating the effects of interventions, although they depend on expert knowledge to define initial causal relationships.

In a similar context, Mehdiyev et al. [15] addressed uncertainty in predictive process monitoring by integrating information systems, machine learnig, and operations research techniques. By using quantile regression forests (**QRF**) and Shapley Additive Explanations (**SHAPs**), they provided a deeper understanding of model uncertainties. This methodology resonates with the findings of Wang et al. [16], who applied deep learning techniques such as **CNN** and **BigRu** to capture dependencies in business processes, improving the prediction of future activities. Both approaches underscore the importance of managing uncertainty and dependencies in data to enhance intervention decisions.

Furthermore, Kotsias et al. [17] integrated deep learning techniques into Business Process Management (**BPM**) through process mining. Their innovative approach combined predictive and prescriptive monitoring using Reinforcement Learning (**RL**) in the banking sector. Methods such as LSTM and Q-learning were employed to predict future states and optimize decisions, highlighting the Inductive Miner, a process discovery algorithm, for its flexibility and scalability. Results showed that the RL model outperformed traditional methods, suggesting future research in RL algorithms and deep learning for complex business environments.

Zahra et al. [18] introduced a prescriptive approach that applies causal inference to estimate the effects of interventions in real time, particularly in the execution of approval request processes. This method adjusts intervention decisions based on user-defined policies and is related to work aiming to improve accuracy in measuring causal effects, as detailed by Pavlos Delias et al. [19]. In this context, doubly robust estimation techniques were employed to mitigate biases and enhance the precision of effect estimates, using simulated process execution data.

Building on the theme of improving predictive accuracy in business processes, Jens Brunk et al. [20] focused on predicting future events by considering context, using dynamic Bayesian networks to model cause–effect relationships. This approach, which combines predictive analytics and machine learning, aligns with the efforts of the previously mentioned articles in seeking to improve the accuracy of predictive process monitoring (**PPM**). However, as in other studies, causality is often assumed based on expert knowledge, which can limit the generalization of the models.

Focusing on the analysis of time series in business processes, Hompes et al. [21] present a technique for identifying causal factors that influence process performance. Their approach, based on Granger causality and combining domain knowledge with advanced clustering and filtering techniques, enables the systematic analysis of event logs to generate causal graphs that reveal underlying relationships between different process variables. The proposed technique includes the creation of a decomposition graph and an inclusion graph, facilitating the discovery of complex interactions between process features and performance metrics.

In summary, there is a need to apply independent and objective causal discovery techniques for anomaly detection in business process monitoring. This approach aims to reduce reliance on expert knowledge and enable a comprehensive interpretation of anomalous event causality, facilitating effective and targeted interventions.

## 4. Proposed Technique

This article presents *CaProM*, an innovative technique for business process monitoring, focused on managing anomalies caused by noise in variables and shifts in the distribution of causal graphs. Unlike previous approaches that rely on expert knowledge [13,14], *CaProM* employs automated causal discovery [22,23], setting itself apart from time series based approaches [21]. It involves independently monitoring causal graphs within each activity of the process flow, providing a deeper understanding that goes beyond effect estimation by identifying the variables that significantly contribute to anomalous events. *CaProM* facilitates the determination of specific actions to address concrete events within the process.

*CaProM* has three stages. First, process events are collected and recorded from project plans, reflecting flow instances that will be executed in the business process. Second, causal learning is carried out by constructing independent directed acyclic graphs (**DAGs**) for each process activity, which represent the dependencies between variables. Third and finally, these DAGs are used to monitor the process, detecting anomalies and critical nodes, based on techniques [10,11].

### 4.1. Project Event Log

**Figure 2:**
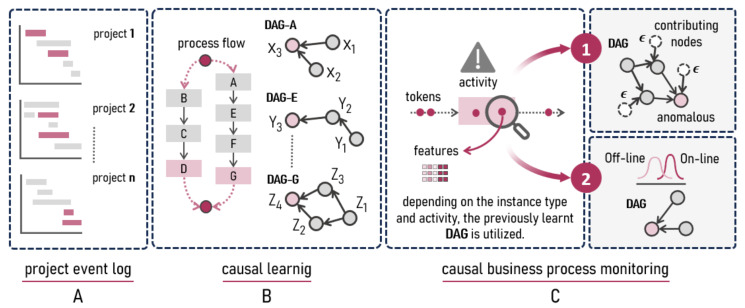
Stages of the proposed *CaProM* technique: event logging, causal learning, and monitoring of anomalies.

*CaProM*’s first stage (Figure 2A) applies process mining techniques to event logs from several projects, to capture the complete model that will be executed. This provides a more accurate representation of operational reality, overcoming the limitations of documented process flows that are often not strictly followed. Event logs for the projects can be sourced from Gantt schedules, and estimates of operational variable usage can be provided by several process roles. Analyzing aggregated traces from the projects allows to identify critical activities and process variants.

For example, software development projects have critical activities proper to software development (e.g., integration and testing), and changes in their requirements impact their execution effort. This first stage is essential to understand the actual execution of processes and uncover patterns and deviations that are not reflected in the official documentation.

In addition, the event log in project planning, as shown in Table 1, includes elements such as the event identifier linked to the project, the planned activity, the timestamp showing the allocated time for completing the activity, the responsible person, and the associated operational variables. These logs detail each stage of the project, allowing for precise tracking of the actual planning for its execution.

### 4.2. Causal Learning

*CaProM*’s second stage (Figure 2B), focuses on the causal analysis of the activities in the process model, obtained from the event logs of multiple projects. Each activity considers a set of operational variables $V^{a}=\left\{X_{1}^{a},\ldots ,X_{n}^{a},Y_{1}^{a},\ldots ,Y_{m}^{a}\right\}$. Using causal discovery algorithms specifically designed for cross-sectional data, DAGs are constructed to represent the causal relationships between these variables, based on simultaneous observations of multiple instances of the activity.

The main goal of this technique is identifying the causal graph that correctly describes the interactions among operational variables, to allowing understanding the causal topology and the specific interrelationships within each process activity. To this end, it employs an algorithm based on the Linear Non-Gaussian Acyclic Model (LiNGAM) that assumes linear relationships and non-Gaussian error terms, thus enabling identification of causal direction even in complex situations with cross-sectional data [24].

(1) $$Y_{i}^{a}=f_{a}\left(\theta_{a},X_{i}^{a}\right)+\epsilon_{Y_{i}}^{a}$$

where $Y_{i}^{a}$ represents the effect variable of activity $a_{i}$ for process instance *i*; $X_{i}^{a}$ is the specific causal variable; $\theta_{a}$ denotes the global parameters; and $\epsilon_{Y_{i}}^{a}$ is the non-Gaussian error term. In addition to LiNGAM, other causal discovery algorithms from the following two main families are employed: *constraint-based* and *score-based methods*.

Constraint-based algorithms, such as the Peter–Clark algorithm (PC), determine the causal structure by conducting conditional independence tests, systematically evaluating whether $X_{1}^{a}⊥\;\;\;⊥Y_{1}^{a}|Z$ for variables $X_{1}^{a}$, $Y_{1}^{a}$, and sets of variables *Z*, thereby determining the existence or absence of edges in the dependency graph ($G_{PC}$). PC starts with a fully connected graph and removes edges based on detected conditional independencies, resulting in a Partially Directed Acyclic Graph (CPDAG).

Score-based algorithms, such as Greedy Equivalence Search (GES), search for the structure that maximizes a scoring function (S(G)). GES operates by performing a greedy search in the space of causal structures, finding $G^{*}=\arg\max_{G}S\left(G\right)$, where $G^{*}$ is the optimal graph according to the scoring function.

**Figure 3:**
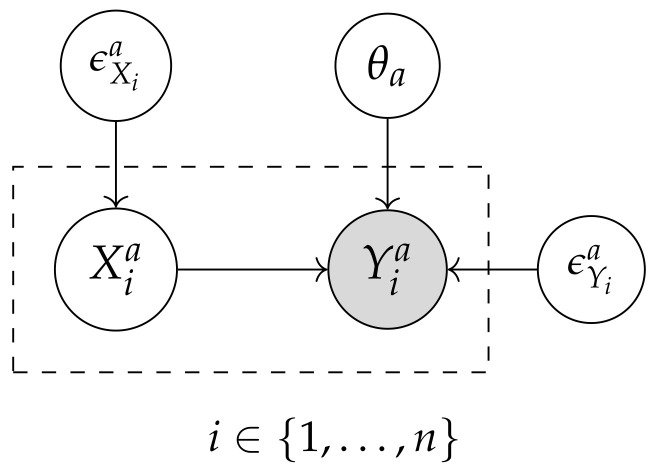
Generalized causal graph illustrating the causal relationships between operational variables, where $X_{1}^{a}$ has a causal effect on $Y_{1}^{a}$, situated in activity *a* of the business process.

Figure 3 illustrates the generalized causal model derived from the causal discovery algorithms [22,23]. This representation explicitly captures the global parameters ($\theta_{a}$) associated with the effect variables ($Y_{i}^{a}$), as well as the error terms ($\epsilon_{X_{i}}^{a}$ and $\epsilon_{Y_{i}}^{a}$), allowing LiNGAM to exploit non-Gaussianity to infer causal direction. In the same graph, the PC and GES algorithms integrate $\theta_{a}$ into their methodologies; PC implicitly utilizes $\theta_{a}$ in the conditional independence tests to determine the dependency relationships between the effect variables ($Y_{i}^{a}$), while GES optimizes a scoring function that incorporates $\theta_{a}$ to evaluate and select the most suitable causal structure from the space of possible graphs.

The causal model for the variables involved in each business process activity can be expressed in terms of their joint probability as follows:

(2) $$P\left(V^{a},\theta_{a}\right)=P\left(\theta_{a}\right)\prod_{i=1}^{n}P\left(Y_{i}^{a}|X_{i}^{a},\theta_{a}\right)$$

where this probabilistic framework facilitates the integration of various causal discovery algorithms to infer the causal structure and estimate the functional relationships between the operational variables. The factorization of the joint probability reflects the structure of the DAG obtained by the algorithms, with each variable ($Y_{i}^{a}$) depending on its direct causes ($X_{i}^{a}$) and the parameters ($\theta_{a}$). Variables ($X_{i}^{a}$) can be considered exogenous or dependent on other variables within the causal model.

### 4.3. Causal Monitoring

In *CaProM*’s third stage, process flow monitoring is based on the previously established DAG, to identify and examine anomalous events in the operational variables. Figure 2C illustrates the process of comparing the variables of interest with a predetermined threshold. When a variable exceeds the established operational limits, the analysis focuses on the causal variables that contributed to the anomaly. To address this causal analysis, two specific techniques are proposed, as described in the following subsections.

#### 4.3.1. Causal Analysis of Anomalous Attributes

A causal analysis of anomalous attributes is conducted by identifying and understanding causal relationships among sources of atypical behavior, by evaluating the data captured during process planning [10], as documented in project event logs (Section 4.1). Additionally, this technique analyzes how noise present in key variables impacts the metrics of interest, to detect unusual patterns and attribute them to specific causes.

**Figure 4:**
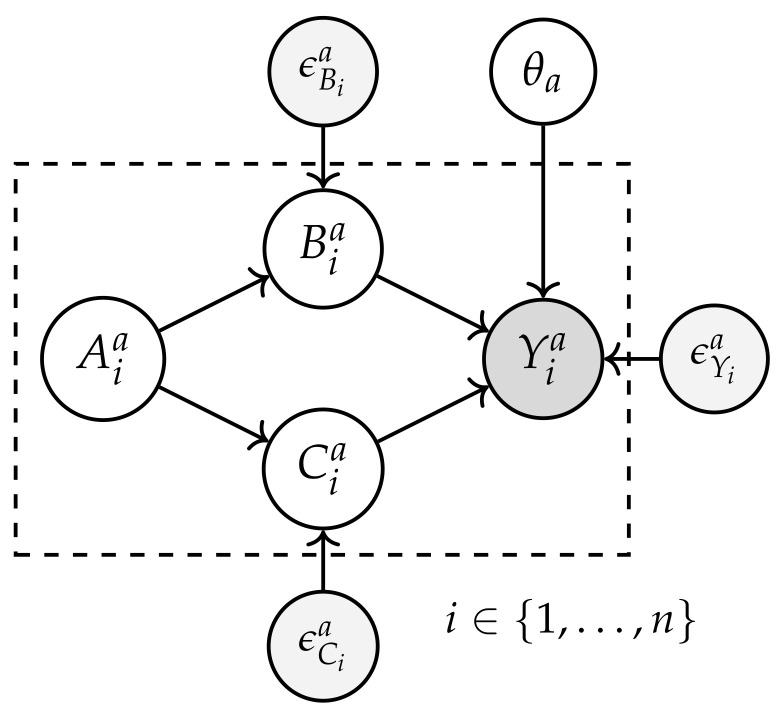
Characterization of the impact of noise on the variables of $Y_{i}^{a}$.

Based on the causal graph topology shown in Figure 4, the perturbations in the error terms ($\epsilon_{B_{i}}^{a}$, $\epsilon_{C_{i}}^{a}$, and $\epsilon_{Y_{i}}^{a}$) are evaluated, and their effects on $Y_{i}^{a}$ are analyzed. Initially, an outlier score for $Y_{i}^{a}$ is established using anomaly detection models such as the difference of means. Subsequently, the contributions of $B_{i}^{a}$, $C_{i}^{a}$, and other causal nodes are quantified, determining how variations in $\epsilon_{B_{i}}^{a}$, $\epsilon_{C_{i}}^{a}$, and $\epsilon_{Y_{i}}^{a}$ influence the probability of $Y_{i}^{a}$ exhibiting anomalous values. This impact is calculated as the probability that $Y_{i}^{a}$ exceeds a threshold ($g(Y_{i}^{a})$) when the values of $\epsilon_{B_{i}}^{a}$, $\epsilon_{C_{i}}^{a}$, and $\epsilon_{Y_{i}}^{a}$ are replaced with random values. it is measured as $-\log P(Y_{i}^{a}\geq g\left(Y_{i}^{a}\right)|$ replace $\epsilon_{i}^{a}$ with random value, where the threshold $g(Y_{i}^{a})$ (e.g., difference between means) determines whether $Y_{i}^{a}$ is anomalous. This logarithmic measure provides a clear assessment of the direct impact of the perturbations.

#### 4.3.2. Causal Attribution of Distributional Changes

**Figure 5:**
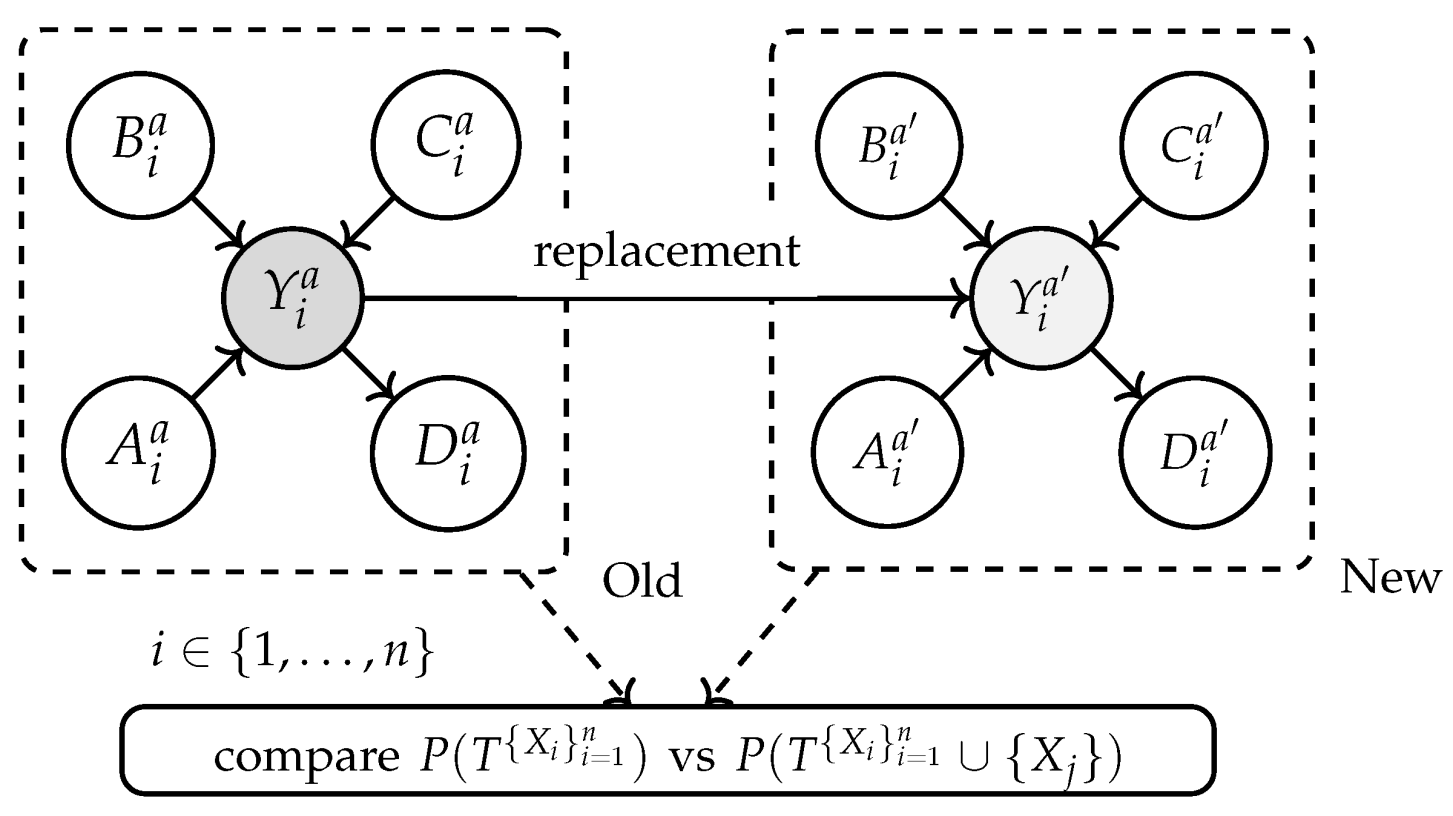
Characterization of distribution change attribution in a DAG for activity $a_{i}$.

The causal attribution of distributional changes aims to identify how changes in variables, such as $X_{i}^{a}=\left\{A_{i}^{a},B_{i}^{a},C_{i}^{a}\right\}$ affect the distribution of the variable of interest ($Y_{i}^{a}$) [11,12]. In Figure 5, the nodes on the left represent the prior data ($P(Y_{i}^{a}|X_{i}^{a})$) obtained from the project event logs, while the nodes on the right show the current data ($\hat{P}\left(Y_{i}^{a^{\prime }}|X_{i}^{a^{\prime }}\right)$) collected during the current process execution of the process (see Equation (2)). This is crucial for business process monitoring, as it allows for an understanding of how changes in operational conditions affect key outcomes.

Unlike previous proposals [13,14,18,20], which focused on estimating causal effects and specific interventions, *CaProM* analyzes variations in the variables distribution. It thus provides a more comprehensive view of how operational environment changes influence the process, capturing not only direct causes but also noise and influences from previous activities. Moreover, the approach is independent of the intervention training, allowing a wider evaluation of operational conditions that could trigger anomalous events.

The process begins with estimation of the conditional distributions of the variables using $P(Y_{i}^{a}|X_{i}^{a})$, where $X_{i}^{a}$ represents the set of direct causal variables that influence $Y_{i}^{a}$; These distributions reflect the planning and expectations based on previous events (project). Next, the previous causal mechanisms ($P(Y_{i}^{a}|X_{i}^{a})$) are systematically replaced with the mechanisms based on the new data ($\hat{P}\left(Y_{i}^{a^{\prime }}|X_{i}^{a^{\prime }}\right)$), which reflect the current reality of process execution. This replacement generates new marginal distributions for $Y_{i}^{a^{\prime }}$, denoted as $\hat{P}\left(T^{X_{n}}\right)$, allowing to observe how these changes affect the variable of interest.

The next step involves comparing the marginal distributions before and after the replacement, specifically $P(T^{{\left\{X_{i}\right\}}_{i=1}^{n}})$ versus $P(T^{{\left\{X_{i}\right\}}_{i=1}^{n}}\cup \left\{X_{j}\right\})$. Here, *T* represents the set of nodes considered in the analysis, and ${\left\{X_{i}\right\}}_{i=1}^{n}$ represents the variables of interest whose distributions are being measured. The notation $\cup \{X_{j}\}$ indicates the inclusion of the distribution of $X_{j}$ with a set of distributions of $X_{i}$, where $X_{j}$ is a new variable or a modification of an existing variable, being evaluated to see how its inclusion or change affects the distribution of the variables of interest.

This analysis helps to identify which node changes (e.g., $A_{i}^{a}$, $B_{i}^{a}$ or $C_{i}^{a}$) are responsible for the observed variations. Understanding the changes in the conditional distributions ($\hat{P}\left(Y_{i}^{a^{\prime }}|X_{i}^{a^{\prime }}\right)$) affect $Y_{i}^{a^{\prime }}$ allows to identify the underlying causes of variations in the performance of process activities. In turn, this enables precise adjustments in operational variables to mitigate issues and optimize system performance.

## 5. Results

This section presents the results obtained by applying the *CaProM* technique described in Section 4, which adapts causal discovery techniques to enable causal learning in the planning and execution stages of a business process. The findings show the efficacy of *CaProM* in detecting and causally interpreting anomalies and distributional shifts during the monitoring of the execution stage. *CaProM* provides insights that go beyond traditional correlations in the analysis of business processes.

### 5.1. Dataset

The validation dataset includes information from 562 projects, each with specific technological and regulatory requirements for a group of banking entities. This dataset provides a comprehensive view of the business process, allowing for the monitoring of planned activities throughout the workflow, observing them in both the planning and execution phases (see Table 2). The stages share the same activities, although they are given different names depending on the phase. For example, an activity labeled *Preliminary Evaluation* during the planning phase may correspond to *Requirement Implementation* during the execution phase, both with the same objective of specifying and fulfilling project requirements.

Additionally, the data follow the structure described in Section 4.1 based on event logging. Key elements include the *Instance ID* to identify each project, the *Activity Name* specifying the actions, the *Execution Times* capturing the duration, the *Roles* assigning responsibilities, and several operational variables.

### 5.2. From Project Plans to Process Map

**Figure 6:**
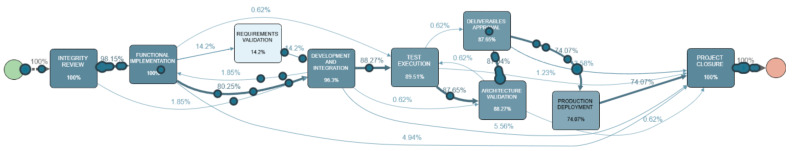
Process flow extracted from the project event logs using Apromore.

The application of *CaProM* (see Figure 2B) allowed to discover the process model from the dataset derived from project planning (Section 4.1). The flow was generated using Apromore, a cloud-based process mining tool that employs an advanced discovery algorithm based on Split Miner [25]. The analysis identified the activities and their interactions, which are represented in Figure 6.

The resulting model provided an accurate visualization of the planning process structure. An activity that captures particular attention from managers is *Test Execution* due to the complexity of project requirements and variability in required hours. This phase, referred to as *Test Design* in project plans, includes testing tasks outsourced by the organization. The variation in the duration of these externalized tasks has a significant impact on other variables of the activity. This finding prompted the identification of causal relationships among variables in order to interpret the effect of outsourced tasks on the activity.

### 5.3. Operational Variables Extracted from *Test Design* for Causal Monitoring in *Test Execution*

Table 3 presents the key operational variables monitored during the *Test Execution* activity. These variables encompass various aspects of the process, such as code coverage, number of meetings, people involved, and test cases. Note that hour-related variables, such as *testing_hours* and *regression_time*, represent estimates of required quantities, not actual measurements over time. These estimates are made by those responsible during the planning phase, and are monitored throughout the test execution.

The analysis confirmed the cross-sectional nature of the data, where each observation represents a unique instance of the process. Although the variables are recorded with a *Time Span* interval based on the project log (see Table 1), multiple execution instances exist at the same temporal point. Addressing temporality under these conditions would require the averaging of data from different simultaneously executed instances, i.e., calculation of an average value ($\bar{X_{t}^{a}}=\frac{1}{n_{t}}\sum_{i=1}^{n_{t}}X_{it}^{a}$) for each instant (*t*), where $n_{t}$ is the number of instances at time *t* in activity *a*. However, this procedure can introduce biases and the loss of crucial information, affecting the estimation of the causal effect ($P(Y_{i}|do\left(X_{i}\right))$). Here, the notation $do(X_{i})$ represents an intervention on the variable $X_{i}$, that is, evaluation an improvement action. Averaging could mask individual variations and temporal dynamics essential for causal discovery, hindering the identification of reliable structures.

Additionally, there is a risk of incurring in Simpson’s paradox, where trends present in subgroups disappear or reverse when data are combined. Consequently, the cross-sectional approach was maintained to preserve the integrity of the relationships between variables and ensure the reliability of the results in the causal discovery methods.

### 5.4. Interpretability with Causal Graphs

The causal learning process shown in Figure 2B was implemented using three algorithms: LiNGAM, PC, and GES (Section 4.2). The application of these diverse methods enabled an exhaustive search for possible causal structures in the dataset, exploring different aspects of the relationships between variables.

To assess the consistency and significance of the causal relationships identified by the algorithms, a measure based on the entropy associated with each arc ($X_{i}^{a}\to Y_{i}^{a}$) was used. After applying the algorithms to each bootstrap sample ($S^{\left(1\right)},S^{\left(2\right)},\ldots ,S^{\left(m\right)}$) yield causal graphs ($G^{\left(1\right)},G^{\left(2\right)},\ldots ,G^{\left(m\right)}$). For each identified arc ($X_{i}^{a}\to Y_{i}^{a}$), its probability was calculated as ($p_{X_{i}^{a}\to Y_{i}^{a}}=\frac{1}{m}\sum_{b=1}^{m}\delta_{X_{i}^{a}\to Y_{i}^{a}}^{\left(b\right)}$), where $\delta_{X_{i}^{a}\to Y_{i}^{a}}^{\left(b\right)}=1$ if the arc $X_{i}^{a}\to Y_{i}^{a}$ is present in the graph $G^{\left(b\right)}$, and =0 otherwise.

The entropy $H(X_{i}^{a}\to Y_{i}^{a})$ associated with each arc ($X_{i}^{a}\to Y_{i}^{a}$) was calculated as $-\left[p_{X_{i}^{a}\to Y_{i}^{a}}\log p_{X_{i}^{a}\to Y_{i}^{a}}+\left(1-p_{X_{i}^{a}\to Y_{i}^{a}}\right)\log\left(1-p_{X_{i}^{a}\to Y_{i}^{a}}\right)\right]$, where $p_{X_{i}^{a}\to Y_{i}^{a}}$ is a Bernoulli random variable. A low value of *H* indicates that the presence or absence of the arc is consistent across the bootstrap samples, suggesting greater reliability and significance in the inference of that causal relationship. This approach allows to quantify the uncertainty associated with each arc, providing a solid foundation for the interpretation of the dependencies among the operational variables identified by the algorithms.

**Figure 7:**
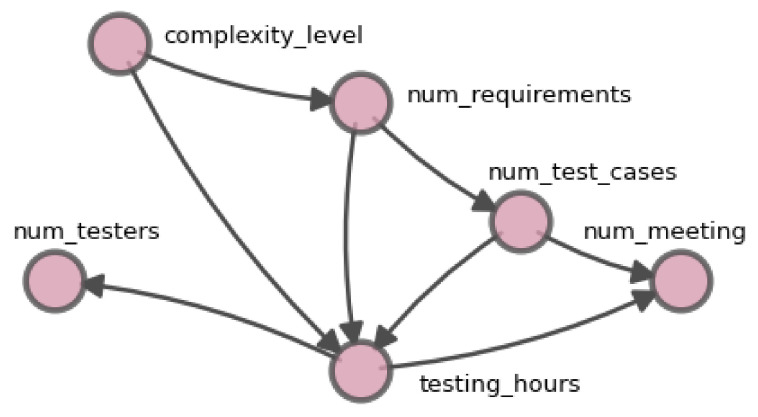
**DAG** extracted in the *Test Design* activity and monitored in the *Test Execution* activity.

Analyzing the extracted DAG (see Figure 7) allows to identify several significant causal relationships among the operational variables. The complexity level influences the number of requirements, which affects the number of test cases which in turn determines the necessary testing hours. Furthermore, the number of testers directly impacts the testing hours which in turn influence the number of required meetings. These connections allow to understand how the monitored variables interact in the test execution process, reflecting the underlying causal structure identified through the applied algorithms.

**Figure 8:**
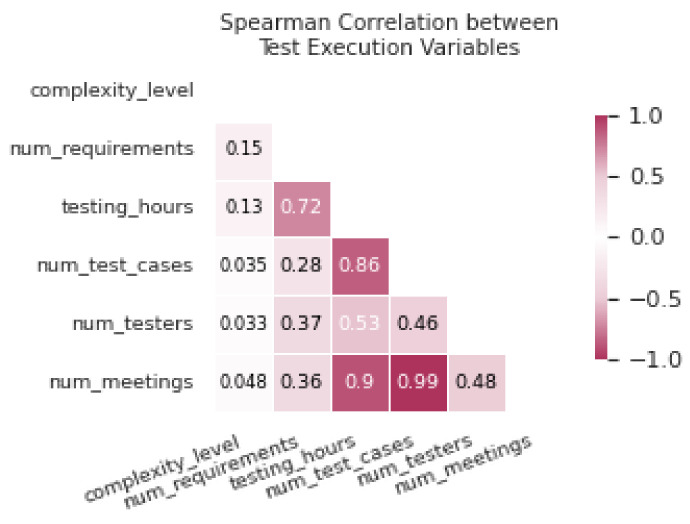
Correlations of the **DAG** variables in the *Test Execution* activity.

According to the correlation heat map presented in Figure 8, which uses Spearman’s correlation due to the nonlinearity present in some data relationships, it is observed that not all variables in Table 3 exhibit significant correlations, although some demonstrate direct causal relationships. For instance, between *num_requirements* and *num_test_cases*, a moderate correlation of 0.28 is observed, despite there being a direct causal relationship between them. Similarly, variables such as *testing_hours* and *num_testers* show a positive correlation of 0.53, supporting their causal relationship. However, in the case of *complexity_level*, a causal influence is identified on the number of requirements, but the correlation is low (0.15), illustrating that a low correlation does not imply the absence of causality.

### 5.5. Explainability of Anomaly Attribution through Causal Graph

**Figure 9:**
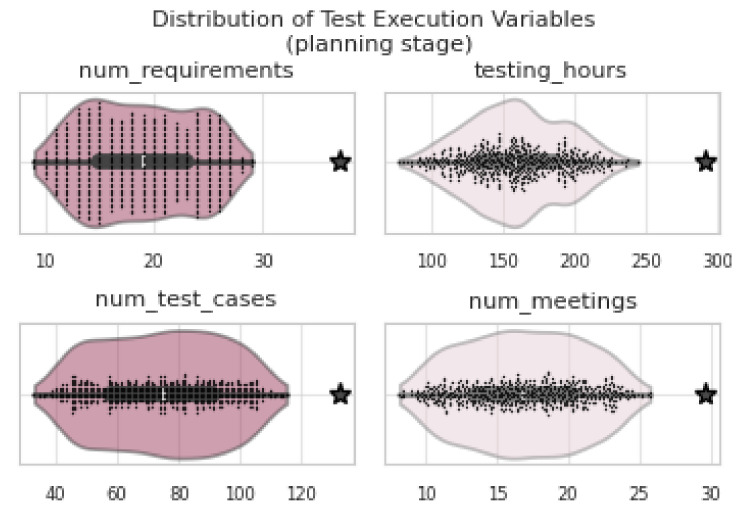
The stars (***★***) represent outlier values in *Test Execution* variables that exceeded (3σ).

At this stage, as described in Section 4.3.1, the causal graph obtained during the planning phase (see Figure 7) was monitored throughout the execution. A threshold of 3σ (*three standard deviations*) was applied to detect potential anomalies in key variables, allowing for the identification of values that significantly deviated from expected outcomes. Variables exceeding this threshold were flagged as anomalous events, aiding in the identification of specific deviations during project execution. Among the cases identified using this approach are the deviations listed in Table 4. The values recorded during execution, when compared to the distribution estimated from the planning phase (see Figure 9), revealed significant deviations in several variables. Specifically, *num_requirements* (37), *testing_hours* (290.9), *num_test_cases* (132), and *num_meetings* (29.52) exceeded the threshold of three standard deviations. These anomalies, detected through the monitoring of the causal graph, indicate that project execution deviated considerably from the original plan, likely due to unforeseen complexities or changes in scope.

The violin plots presented in Figure 9 clearly show that these anomalous values were situated outside the estimated distributions.

At this analysis stage, the anomaly attribution DAG-based algorithm (see Figure 7) identified the causal topology for the observed value of *num_meetings* ($Y_{i}^{a}$). This enabled monitoring of the relationships among variables during the DAG execution, evaluating perturbations in the error terms ($\epsilon_{i}$) for each variable, and quantifying the perturbations impact on the anomalous behavior of $Y_{i}^{a}$.

Decomposing the system to understand how interactions among upstream nodes impact to the final outcome is crucial to calculate the probability that $Y_{i}^{a}$ exceeded the 3σ reference threshold.

**Figure 10:**
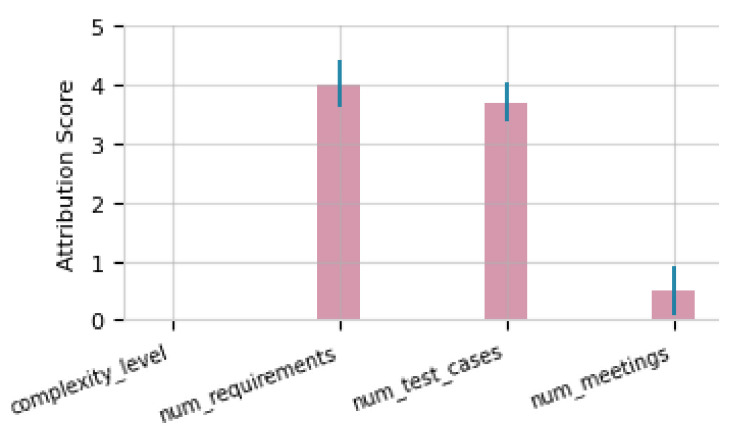
Attribution score of the anomalous values.

The score attribution graph Figure 10 show how variables *num_requirements* (37) and *num_test_cases* (132) had a causal impact on the observed anomaly in *num_meetings* (29.52), exceeding the deviation thresholds. The direct impact of perturbations in these key variables showed that *num_requirements* achieved an attribution score close to 4.5, reflecting its predominant influence. *num_test_cases* had a score close to 4, also a significant impact albeit somewhat lower. In contrast, *complexity_level* (2) and *num_meetings* presented low scores of around 0 and 0.5, respectively, indicating that their influence was marginal and mediated by other variables.

The anomaly attribution analysis focused on the observed behavior of *num_meetings* ($Y_{i}$ = 29.52). By applying attribution techniques, perturbations in the error terms ($\epsilon_{H_{i}}$, $\epsilon_{C_{i}}$, and $\epsilon_{Y_{i}}$) were evaluated to examine how these variations affected the probability of $Y_{i}$ exhibiting an anomalous value. In the context of the causal graph, upstream variables such as *testing_hours* ($H_{i}$ = 290.90) and *complexity_level* ($C_{i}$ = 2) were considered. However, their contributions to the anomaly were significantly smaller than those of *num_requirements* ($R_{i}$ = 37.0) and *num_test_cases* ($N_{i}$ = 132). This analysis allowed to quantify the contribution of variations in $\epsilon_{i}$ to the anomalous behavior of *num_meetings*, by calculating $-\log P(Y_{i}\geq g\left(Y_{i}\right)|\mathrm{replacement}\;\mathrm{of}\;\epsilon_{i}\mathrm{with}\;\mathrm{random}\;\mathrm{values})$, where $g(Y_{i})$ represents the reference threshold to determine whether $Y_{i}$ is anomalous.

The results confirmed that *num_requirements* ($R_{i}$ = 37.0) was the primary trigger, generating a cascade effect that significantly impacted other variables connected to its downstream nodes in the *Test Execution* activity during project execution.

### 5.6. Explainability of Distributional Changes through Causal Graph

**Figure 11:**
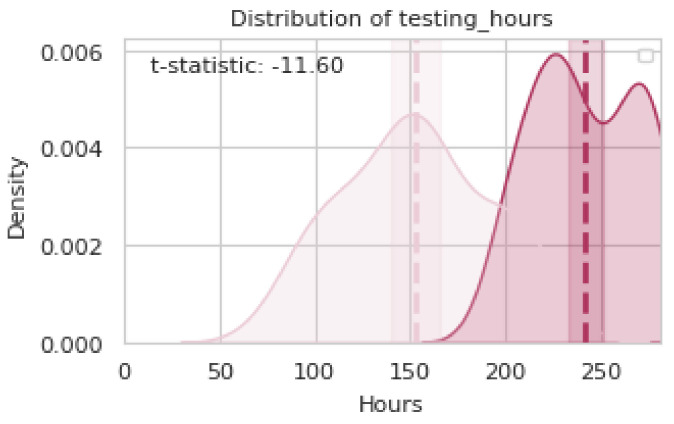
Distribution of *testing_hours* between the 37 Specific and Planned Cases.

An analysis of distribution changes in a representative subset of the data showed a significant discrepancy in *testing_hours* between the planned and observed values (see Figure 11). The planned distribution had a mean close to 150 h but the observed distribution (*execution*) had a central concentration around 250 h. This shift was confirmed with a *t*-statistic of (−11.60), evidencing a statistically significant difference in the analyzed sample. Further analysis showed that the testing times executed in this subset considerably exceeded the planned estimates, indicating a potential impact on system performance. The technique (Section 4.3.2) quantified this deviation, highlighting the need to identify the underlying causes in the DAG [11].

The application of the distributional causal analysis technique to *testing_hours* involved comparing the conditional distributions of $P(Y_{i}^{a}|X_{i}^{a})$ (*planning*) and $\hat{P}\left(Y_{i}^{a}|X_{i}^{a}\right)$ (*execution*). *testing_hours* ($Y_{i}^{a}$) was identified as the variable of interest, with parent nodes ($X_{i}^{a}$) including *num_test_cases*, *complexity_level*, *num_requirements*, and *num_meetings*. $P(Y_{i}^{a}|X_{i}^{a})$ was estimated from the planning data and replaced by $\hat{P}\left(Y_{i}^{a}|X_{i}^{a}\right)$ using the data observed during execution. This produced a new distribution ($\hat{P}\left(T^{X_{n}}\right)$), which allowed for observation of how variations in the parent nodes affected the total testing time. The KL divergence ($D_{KL}\left(P\left(Y_{i}^{a}|X_{i}^{a}\right)\|\hat{P}\left(Y_{i}^{a}|X_{i}^{a}\right)\right)$) quantified the impact of these changes, allowing for the identification of the main causes of the discrepancy in *testing_hours*.

The analysis results show that *num_test_cases* was the primary cause of the distribution shift of *testing_hours*, with an observed difference of 250 h during execution. The Comparison of distributions confirmed a significant alteration, highlighting discrepancies between the planning and execution of the projects.

**Figure 12:**
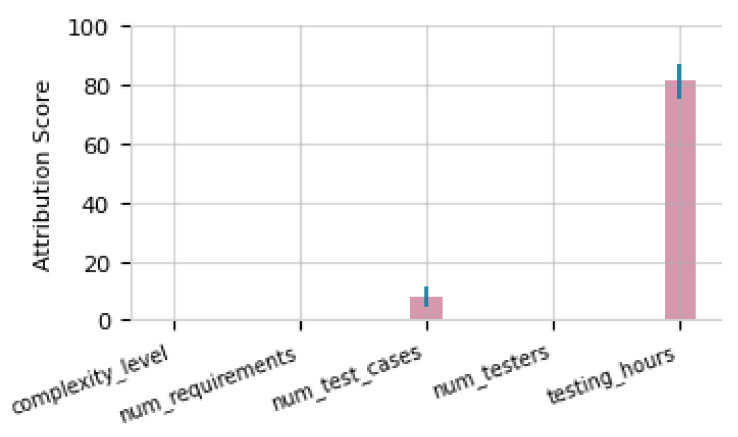
Attribution Score for the Distribution Change of *testing_hours*.

The bar chart reflects the contribution of each variable to the change in the distribution of *testing_hours* (Figure 12). *num_test_cases* has an attribution score close to 10, indicating a causal responsibility with respect to the observed change. In contrast, other variables *complexity_level*, *num_requirements*, and *num_testers* present attribution scores near zero, confirming that their effects on the distributional shift were minimal. These results highlight that the most significant change occurred in *testing_hours*, driven primarily by the causal factors represented in *num_test_cases*, while the other variables had a marginal influence.

## 6. Discussion

The study of the process flow (see Figure 6) demonstrated the way in which activities recorded in the projects can be modeled as a business process flow using process mining algorithms. This representation enabled the precise identification of activities and their interactions, emphasizing the importance of the *Test Execution* activity for managers due to the outsourcing of tasks. The combined application of causal discovery algorithms, with connections evaluated through entropy (Section 5.4), facilitated the creation of a reliable causal graph, represented as a DAG (see Figure 7), which showed the causal relationships between operational variables (Section 5.3).

The anomalous values (Table 4) identified in key process variables were linked to the proposed research questions (see Section 2.3). The resulting causal graph enabled the examination of these questions through the analysis of causal pathways. The path of *num_requirements* →*testing_hours*→ *num_meetings* showed the propagation of anomalies in the system ([rq: rq2]RQ.2). The graph facilitated the assessment of how specific variables influenced the observed anomalies ([rq: rq1]RQ.1), for example, the effect of *num_requirements* on *testing_hours*. This approach also allowed for the analysis of changes in the distribution of *testing_hours* ([rq: rq3]RQ.3 and [rq: rq4]RQ.4) in a subset of 37 projects, revealing how changes in causal relationships impacted the *Test Execution* activity during process execution.

Unlike previous studies [13,14,19,20], *CaProM* incorporated causal discovery into process planning. It not only identified causal relationships but also determined the influence of operational variables on anomalies and distributional changes in process activities. The integration of causality into project planning allowed addressing the dynamic nature of the environment, facilitating adjustments based on causal evidence. The causal analysis employed cross-sectional data, avoiding the need to average multiple time instances and reducing potential biases. Unlike time-series methods (e.g., [21]), this approach better preserves the relationships among variables. By avoiding issues with simultaneous data (Section 5.3 and Section 5.4), the causal discovery is beneficial to process managers, minimizing spurious relationships and providing actionable information for decision making.

The results in (Section 5.5 and Section 5.6) show that causal anomalies were identified in the *Test Execution* activity during the execution phase, which was monitored because of the outsourced tasks. The attribute anomalies in *num_requirements* (37) and *num_test_cases* (132) exceeded the 3σ threshold, affecting execution and contributing to the anomaly in *num_meetings* (29.52). In terms of distributional anomalies, *testing_hours* exhibited a significant shift (planned average: 150 h; observed: 250 h). Causal analysis attributed this change primarily to *num_test_cases*, with a score close to 10, while *complexity_level*, *num_requirements*, and *num_testers* made minimal contributions.

This work still has some limitations. The primary limitation (and requirement) is the fundamental need for detailed records of process planning, which are crucial for causal learning; this restricts its applicability in contexts where planning data are unavailable or incomplete. Also, the omission of organizational external factors may affect the accuracy of the causal relationships. Finally, the use of large data volumes (i.e. event logs) introduces computational challenges of its own.

## 7. Conclusions

This article has presented applied research aimed at improving the monitoring and interpretation of anomalous events in business process management through the early integration of causal discovery techniques. It introduced *CaProM*, an approach that uses knowledge of causal structures from initial project stages to enable monitoring and greater explainability during process execution. The key findings of this study are:

- Successful adaptation of causal discovery algorithms (LiNGAM, PC, and GES) to identify causal structures in 562 activity flow plans during the planning phase (see Section 5.1), revealing relationships not evident with traditional methods;
- Practical application of causal attribution techniques for anomalies and distributional changes, using the causal knowledge obtained during planning to more accurately interpret anomalous events during the execution of business process; and
- Demonstration of how integrating causal learning from the planning phase can significantly enhance the interpretability and explainability of anomalies during process execution, enabling a more informed and effective response.

*CaProM* advances the adaptation of causal learning techniques for monitoring business processes, promoting a shared understanding of project managers and process managers. It enhances analysis during execution, drawing on causal knowledge obtained during planning, and providing a solid foundation for the causal detection and explanation of anomalies and for making informed decisions. *CaProM* introduces a new way to anticipate causal scenarios in complex processes, enabling organizations to monitor them more effectively and make informed decisions during their execution. By integrating a causal view from the planning phase, managers can detect anomalies and understand their root causes using existing causal knowledge. Additionally, *CaProM* can interpret distributional changes to enable dynamic process adaptation, providing a competitive advantage in dynamic business environments that demand rapid detection, explanation, and response to changes in process patterns [26].

Future research will address unobserved confounding variables and explore the grouping of variables by process instance type, which could generate multiple **DAGs** per activity. This approach would offer a more detailed view of operational dynamics in numerous business contexts.

## Figures and Tables

**Table 1:** Project event log.

Project ID	Activity	Time Span	Role_1_	Feature_1_	Feature_2_	…	Feature_*n*_
proj._1_	A	350.2 ms	role._1_	-	feat._2_	…	feat._*n*_
proj._1_	B	270.2 ms	role._2_	feat._1_	feat._2_	…	-
proj._1_	C	450.5 ms	role._4_	-	feat._2_	…	feat._*n*_
proj._2_	A	510.4 ms	role._5_	feat._1_	-	…	feat._*n*_
proj._2_	B	350.8 ms	role._1_	feat._1_	feat._2_	…	-
…	…	…	…	…	…	…	…
proj._*n*_	A	230.4 ms	role._5_	-	feat._2_	…	feat._*n*_
proj._*n*_	B	130.6 ms	role._2_	feat._1_	-	…	feat._*n*_
proj._*n*_	C	310.2 ms	-	feat._1_	feat._2_	…	-

**Table 2:** Process flow activities.

No.	Planning Activities	Execution Activities
1	Requirements Analysis	Integrity Review
2	Preliminary Evaluation	Requirements Validation
3	Functional Specification	Functional Implementation
4	Technical Design	Development and Integration
5	Test Design	Test Execution
6	Architecture Design	Architecture Validation
7	Project Approval	Deliverables Approval
8	Implementation Planning	Production Deployment
9	Request Closure	Project Closure

**Table 3:** Variables in the *Test Execution* activity.

No.	Variable	Description
1	test_coverage	Percentage of code or requirements covered by tests
2	num_meetings	Hours dedicated to meetings for coordination, clarification, and scope changes
3	testing_hours	Hours dedicated to testing
4	num_testers	Number of people involved in testing
5	regression_time	Time spent on regression testing after changes
6	complexity_level	Complexity level of the requirement
7	num_test_cases	Number of cases in the test plan
8	defects_found	Number of defects identified during testing
9	automation_level	Percentage of test cases that are automated
10	num_requirements	Number of associated functional requirements

**Table 4:** Anomalous values of a *Test Execution* requirement versus its *Test Design* planned value.

Variable	Anomalous Values
complexity_level	2.0
num_requirements	37.0
testing_hours	290.90
num_test_cases	132.0
num_testers	6.0
num_meetings	29.52

## Data Availability

The data used in this study are not publicly available due to confidentiality agreements and legal restrictions with the company that provided the information.
